# 2006 to 2019 Story; percutaneously implantable aortic valve prototypes

**DOI:** 10.1186/s13019-021-01597-6

**Published:** 2021-08-06

**Authors:** Constantinos Zervides, Ornella Nohra, Gabriel Hunduma, Neil Wild Thomas, Ramy Samia

**Affiliations:** University of Nicosia Medical School, University of Nicosia, 21 Ilia Papakyriakou, 2414 Engomi, Nicosia, Cyprus

**Keywords:** Aortic valve, Prototype(s), Percutaneous implantation, Cardiovascular implant

## Abstract

**Aims:**

A review was conducted on the composition, advantages and limitations of available aortic valve prototypes to create an ideal valve for percutaneous implantation.

Patients

Patients with multiple comorbidities who cannot withstand the risks of open cardiac surgery.

**Methodology:**

The search was performed using online databases and textbooks. Articles were excluded based on specific criterion.

**Results:**

Ten prototypes created between 2006 and 2019 were found and reviewed. The prototypes had a set of advantages and limitations with their characteristics coinciding at times.

**Conclusions:**

The ideal percutaneously implantable aortic valve should have minimum coaptation height, zero folds in the leaflets, minimum valve height, minimum leaflet flexion and three leaflets. It can be composed of biological or synthetic material, as long as it provides minimal risk of thrombosis. However, more studies are needed to ensure other ideal parameters.

## Introduction

Transcatheter aortic valve replacement (TAVR) has a significant role in reducing mortality in high-risk patients who cannot undergo open surgery [1]. Over the past 20 years, improvements in anatomical knowledge and valve design have secured TAVR a place in clinical practice [2].

### Role of TAVR in modern medicine

The burden of aortic valvular disease varies greatly across socioeconomic environments, with degenerative aortic valve calcification being the predominant cause in high-income countries, and infectious disease being a significant cause in developing nations [3–6]. Because of this degenerative aetiology in high-income countries; aortic valve disease prevalence increases exponentially with age and is commonly associated with multiple comorbid conditions [3–5].

The current standard treatment for severe aortic stenosis, in patients of high or intermediate risk, is TAVR [2, 7, 8]. Recent studies have demonstrated potential for TAVR to replace open surgical approaches in low-risk patients [9–11]. However, procedural outcomes are still heavily reliant on operator training and familiarity with the relatively new techniques [9].

The current standard of treatment for aortic regurgitation, a significant sequelae of infectious aortic valve disease, is a medical and open surgical treatment [12]. TAVR has become the preferred approach of management in patients not suitable for surgery, leading to improved designs of better performing percutaneously implantable valves [12–14].

### History of aortic valve replacement

The transcatheter approach for aortic valve implantation first came to light in the early 1990s with work by Dr Alain Cribier and has rapidly graduated from restricted compassionate use to the clinical standard it is today [2, 15]. Animal studies by Anderson and colleagues in 1992 demonstrated the successful stented implantation of valves in various cardiac sites using a transcatheter approach [16]. Anderson and colleagues utilised a hand-made porcine valve that was contained within a metallic mesh [16].

The first finalised device created for transcatheter aortic valve replacement was developed by Dr Cribier in the late 1990s, with its first animal studies in sheep being performed in 2000 [15, 17]. This design involved a stainless-steel stent, 23 mm in diameter and 17 mm in height. Within this, a tri-leaflet valve was mounted. Initially, this valve was constructed from polyurethane but was later changed to the bovine pericardium [15]. When prepared for delivery, the entire device was compatible with a 24F introducer sheath. The first successful implantation of this design was performed in a sheep via a brachiocephalic approach [17].

The first successful human implantation of this design was performed on April 16, 2002, by Dr Cribier and colleagues [15, 18]. The patient was 57 years old, with severe aortic stenosis, cardiogenic shock, left ventricular dysfunction, and multiple comorbidities [18]. The transcatheter aortic valve replacement was proposed as a last resort option and was performed through an unplanned antegrade trans-septal approach [15, 18].

Following its first successful human application, feasibility trials on the Cribier design were restricted to compassionate use [15, 19]. For these trials, the valve structure was modified again, to utilise an equine pericardium material. These early trials confirmed the feasibility of the anterograde trans-septal approach for transcatheter atrial valve replacement [15, 19].

Following this proof of feasibility, Edwards Lifesciences developed a new design for the TAVR application [15]. This new design was coined the Edwards-SAPIEN valve. The design involved a tri-leaflet bovine pericardium valve mounted within a balloon-expandable stainless-steel stent. The pericardium was pre-treated in order to decrease calcification following implantation. This new design was available in two sizes, 23 mm and 26 mm, to allow for more accurate fitment in different sized patients [15, 20].

In 2005, the self-expanding CoreValve™ aortic valve prosthesis demonstrated promise with its first-in-man use [21]. The self-expanding stent was constructed using a laser-cut nitinol tube, within which a bovine valve was mounted [21]. The self-expanding nature of this valve was believed to potentially reduce paravalvular leakage, as its expansion would continue over time, unlike its balloon-expanding counterparts [21].

In 2007, the Edwards-SAPIEN prosthesis (originally known as Cribier-Edwards) was further developed. This design included a pericardial xenograft mounted on a stainless-stent and was again available in both 23 mm and 26 mm diameters [22]. The lower inflow portion of this valve was covered with a polyethylene terephthalate (PET) cloth, and the valve was mounted in a suture-less fashion. This design allowed for the antegrade delivery of the valve [22].

A trial on the initial 50 patients treated with the Edwards-SAPIEN prosthesis was performed by Walther and colleagues [23]. Patients in this trial had severe aortic stenosis and high perioperative risk. All patients were aged 75 years or older and had an Aortic annulus diameter of 24 mm or less. This trial demonstrated that the antegrade approach reduced 30-day mortality rates and improved implantation success rate compared to the transfemoral approach. Walther and colleagues noted the value of the valve-in-a-valve concept, which allowed placement of new valves within an initial stent in the case of valve degeneration. The Edwards-SAPIEN prosthesis was limited by its requirement for a well-equipped hybrid operating theatre with high-quality fluoroscopy and transesophageal echocardiography capabilities. This study was limited by the inability to perform a truly randomised study comparing the efficacy and benefits of the transapical and femoral techniques [23].

For the benefit of the reader, a more detailed history of the aortic valve is presented in the paper by Figulla et al. [24].

### Anatomy of the aortic valve and construction of prothesis

The aortic valve is situated between the left ventricle and the aorta and allows blood to flow in one direction without going back. The aortic valve is made up of three leaflets, each shaped like a half-moon. The leaflets represent the mobile part of the valve. The valve is also made up of three sinuses which represent dilations of the aortic base and whose collective diameter is almost twice as that of the aorta. Additionally, sinuses play an essential part in the procedure of valve closure [25–29]. It is essential to keep in mind that the leaflet-sinus combination plays a role in how stresses are equally distributed in the valve and that the leaflet design is optimised to withstand such stress [25–27, 29, 30].

The leaflets are structured to withstand stress optimally in a cylindrical shape which allows for reversal of curvature in both opening and closing [25]. Through the cylindrical shape, easier reversibility can be achieved with one leaflet compared to having two leaflets [25].

To construct the valve, ten parameters were taken into consideration; radius of the base and commissures, height of the valve, angle of free edge to the plane through the three commissures, angle of the bottom surface of leaflet to the plane through the three commissures, coaptation height at the centre, commissural height, length of the leaflet free edge, length of the leaflet in the radial direction, sinus depth and sinus height [25–27]. These dimensions do not stay constant; they change as a function of aortic pressure and time during each heart cycle [25]. The change in function shows that the aortic valve has a dynamic configuration [25]. For example, the radius of the base and the bottom surface angle slightly increases in response to a rise in pressure however the valve height remains consistent, and the coaptation height remarkably decreases [25].

Five parameters are of main focus when designing an aortic valve. They are the radius of the base, radius of the commissures, valve height, commissures height and the angle of the open leaflet to the vertical known as Beta [25, 26, 31].

Also, it is vital to know the composing features of a valve. Firstly the leaflet must be cylindrical. The free edge of the open leaflet must lie in a plane passing through the three commissures, and part of the line of the leaflet connection must be vertical. Lastly the leaflet’s attachment residual line should also lie on a plane intersection, enabling the leaflet reflection to produce a closed valve [25].

There are specific optimal values for the parameters which act as reference values [25]. The radius of the base should be 10 mm in length, while the radius of commissures 8.3 mm and the valve height 11.7 mm. The commissure height should be 1.8 mm and the angle Beta 5.6 degrees [25]. It is important to note that these measurements may vary with each valve design [25].

### Objective

Part of the review included a section on the anatomy of the aortic valve and a history and role of TAVR. This is purely for coherence and further benefit of the reader. However, the main objective of this review was to analyse a selected range of developments of transcatheter aortic valve implants over the past 15 years, whilst detailing strengths and weaknesses of each design.The review also provides an outline of how an efficient and effective prosthetic design is produced when using the optimal geometric measurements of the native aortic valve.

## Methodology

A systematic review was conducted on the historical perspective of heart valve developments, the geometrical importance of aortic valves, and ten of the most recently designed percutaneous prosthetic aortic valve prototypes including prototypes approved for clinical use.

An electronic search was performed using Google Patents, to access the Google Patents Public Datasets, PubMed Central database in combination with Google Scholar, and an online medical device exhibition webpage called Medical Expo. The search was run between April and May 2020 and included all literature that was published on Google Patents and Google Scholar before May 31, 2020, in English. A separate search was performed on the medical exhibition page for current aortic valves that were approved for clinical use. The articles collected were stored on a hard-drive for later access and citing.

An extensive search strategy on Google Patents was performed using one or a combination of the following three terms: prosthetic heart valve, prosthetic aortic valve or percutaneous aortic valve. No date limitations were set. The search strategy on Google Scholar involved a combination of the following terms: TAVR, history, heart valve replacement, aortic valve replacement, first valve implant, history of valves, and percutaneous valve implant history. Furthermore, the search on the online medical device exhibition webpage was limited to aortic valves of any date, and the reference book titled 'The Aortic Valve' by Mano J. Thubrikar was extensively used [25].

The exclusion criteria used in the prototype selection include tricuspid, mitral or pulmonary prosthetic valve designs and aortic prosthetic valves constructed for open repair purposes. Non-patent articles were excluded from the prototype and clinically used valve designs.

### Data collection

Suitable publications were collected in a standardised manner, using the following: title, description and exclusion criteria. Data were extracted independently from each eligible peer-review, patent article and the pre-selected reference textbook.

### Data items

For the historical perspective of valve development, the date of invention for each of the valves, the first model formed and the date of its first use in clinical practice were extracted. For the anatomical description of the aortic valve, the optimal geometrical coordinates required to design an efficient and effective valve were extracted. For the prototypes or clinically approved valves currently designed, data from patent-only articles within the criteria of percutaneous use were extracted.

### Outcomes and prioritisation

The primary outcome was to produce a timeline of the historical developments of prosthetic valves, together with a list of the available prototypes and clinically approved prosthetic aortic valves used in the percutaneous approach.

## Results

Patent US20060271172A1, titled Minimally Invasive Aortic Valve Replacement, comprises a valve body with a leaflet apparatus (using polytetrafluoroethylene (PTFE), bovine pericardium, or native porcine valve material), a supporting stent (metallic frame comprised from a nickel-titanium alloy known as nitinol or stainless steel), an O-ring assembly surrounding the valve body (comprised of expanded polytetrafluoroethylene, foam, or rubber) a superior and an inferior O-ring (comprised of felt) [32].

**Fig. 1:**
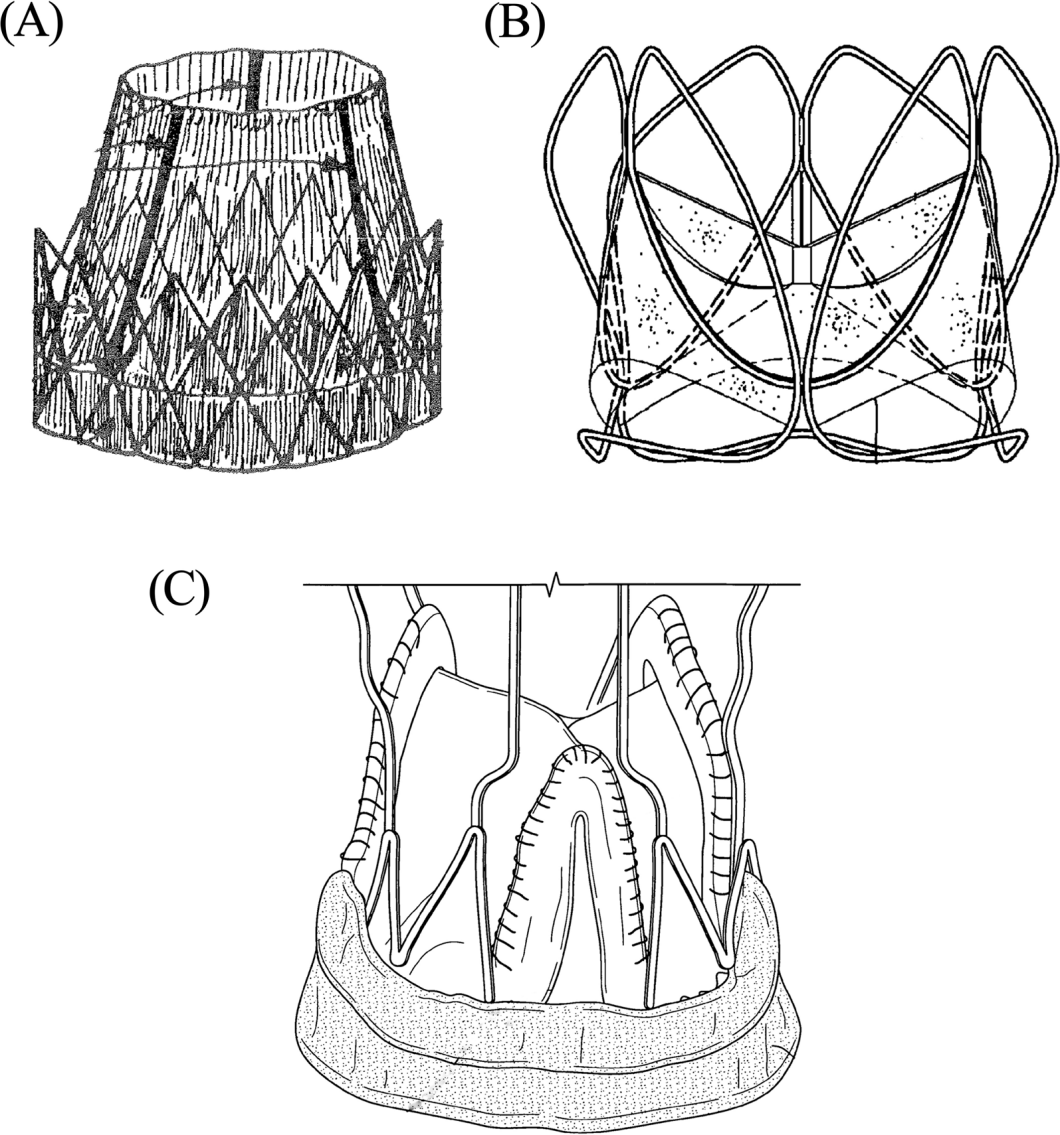
Schematics of different patented valve designs. **A** Prototype US7846204B2—Courtesy of Edwards Lifesciences LLC, Irvine, CA. [33]. **B** Prototype US20120165929A1—Reproduced with permission from UCL Business Ltd. [34]. **C** Prototype US8425593B2—Reproduced with permission from Abbott [35]

Patent US7846204B2 (Fig. 1A), titled aortic valve prosthesis, having natural tissue and an internal cover, comprises a valvular tissue made of a synthetic biocompatible material such as TEFLON or DACRON polyethylene, polyamide, or biological material such as pericardium, porcine leaflets and the like. The frame is a stainless metal structure or a foldable plastic material, made of intercrossing linear bars, preferably rounded and smooth. The frame has to project curved extremities and requires a concave shape to strengthen the insertion and locking of the valve in the deformed aortic orifice [33].

Patent US20120165929A1 (Fig. 1B), titled heart valve prosthesis, comprises a support structure with a framework deformable between an expanded state and a compressed state. It also comprises a flow-control structure, with a support structure, for permitting blood flow in one direction, defining an axial direction of the prosthesis, and for restricting the blood flow in an opposing direction, wherein at least one end of the support structure comprises a plurality of apexes of the framework. The support structure is collapsible into the compressed state by pulling on the apexes, to enable it to be drawn into a sheath in the compressed state, the sheath having an inner radial dimension smaller than the radial dimension of the support structure in the expanded state. The flow control structure of this embodiment can be entirely synthetic, for example, formed from artificial polymeric material, or can be biologically derived, for example, a xenograft of bovine pericardium or porcine pericardium, or a combination of synthetic and biologically derived [34].

Patent US8425593B2 (Fig. 1C), titled collapsible prosthetic heart valve, is a ring-shaped, collapsible and re-inflatable supporting structure prosthetic heart valve. A malleable, sheet-like, leaflet member is set inside the supporting structure, so that a free edge portion of it forms a flexible cord across the interior of the supporting structure. A flap is formed by the material of the leaflet, which is folded to lie, in part, in a cylindrical surface defined by one of the inner and outer surfaces of the supporting structure. Between the supporting structure and the leaflet lies a malleable, sheet-like, buffer material. Buffer material or materials (e.g., polymer sheet or pericardial tissue sheet) are processed and cut to shape. The joint posts of a valve stent can have a bovine jugular or porcine aortic root (or individual leaflets) attached to it [35].

**Fig. 2:**
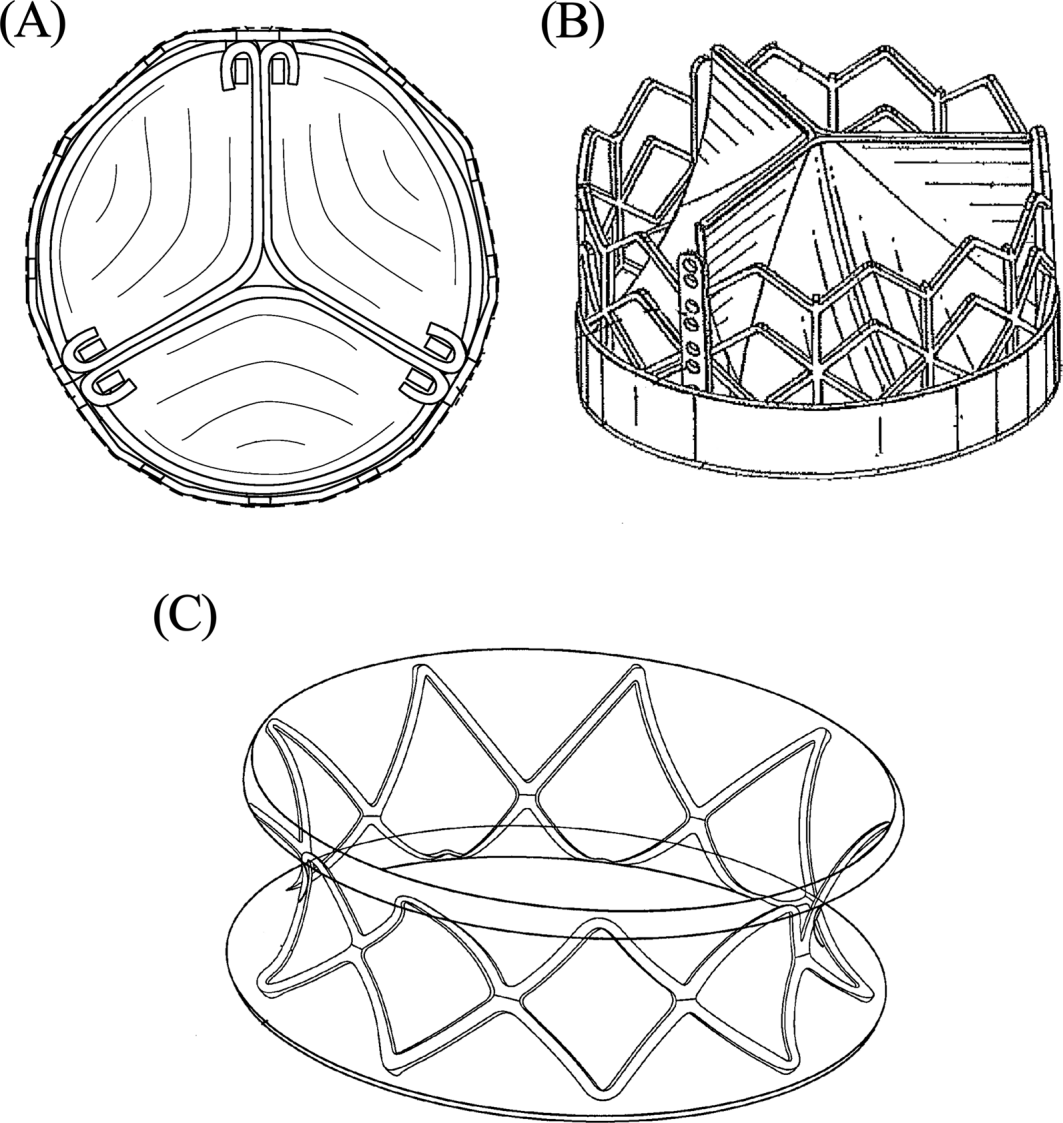
Schematics of different patented valve designs. **A** Prototype US8454685B2—Courtesy of Edwards Lifesciences LLC, Irvine, CA. [36]. **B** Prototype US20140135911A1—Courtesy of Edwards Lifesciences LLC, Irvine, CA. [37]. **C** Prototype US9532868B2—Courtesy of Edwards Lifesciences LLC, Irvine, CA. [39]

Patent US8454685B2 (Fig. 2A), titled low profile transcatheter heart valve, which is clinically used, consists of a radially collapsible and expandable frame, leaflet structure, and a skirt member. The leaflet structure has a scalloped lower edge portion that is positioned inside of and secured to the frame. The frame can be made from nitinol to produce the self-expanding valve function. Alternatively, plastically expandable material that enables crimping of the valve to a smaller profile can also be used [36].

Patent US20140135911A1 (Fig. 2B), titled prosthetic heart valve and method, is composed of a radially crimpable and radially expandable, net-like, annular support frame, and a valve assembly. The valve is made of a conduit that has a directional progression from the inlet towards the outlet. The conduit consists of three flexible cusps, which are crown-shaped cut line and are sutured to the bottom of the frame and around the cut line. The valve assembly can be biological such as natural tissue, pericardial tissue or other types, and alternatively from biocompatible polymers or similar. The support frame contains a proximal and distal portion, with the diameter of the proximal portion being smaller than the diameter of the distal portion [37].

Patent US20140163667, titled stentless aortic valve replacement with high radial strength, comprises first annular support in the assembled valve and would be located at the base or annulus of the valve, as well as second annular support at the commissural region of the valve. The shape of the hoops can be changed to a circular shape, a clover, or a semi-triangular shape to accommodate the natural aortic valve shape. Various forms allow a more uniform fit as well as less radial resistance in crush due to the irregular shape [38].

Patent US9532868B2 (Fig. 2C), titled collapsible-expandable prosthetic heart valves with structures for clamping native tissue, consists of a flexible leaflet structure, an annular structure and perimeter with a changeable length in-between and is adapted for implantation into a native aortic annulus. Linking structures connect the annulus and aortic portions of the valve. These portions also have diamond-shaped cells with each cell containing an upstream apex connected to a downstream apex in a longitudinal direction. The leaflet structure is made of three flexible leaflet sheets composed of either natural tissue, flexible polymer or similar. Each commissure post is partly cantilevered up towards the blood inflow, and the other post down towards the blood outflow [39].

**Fig. 3:**
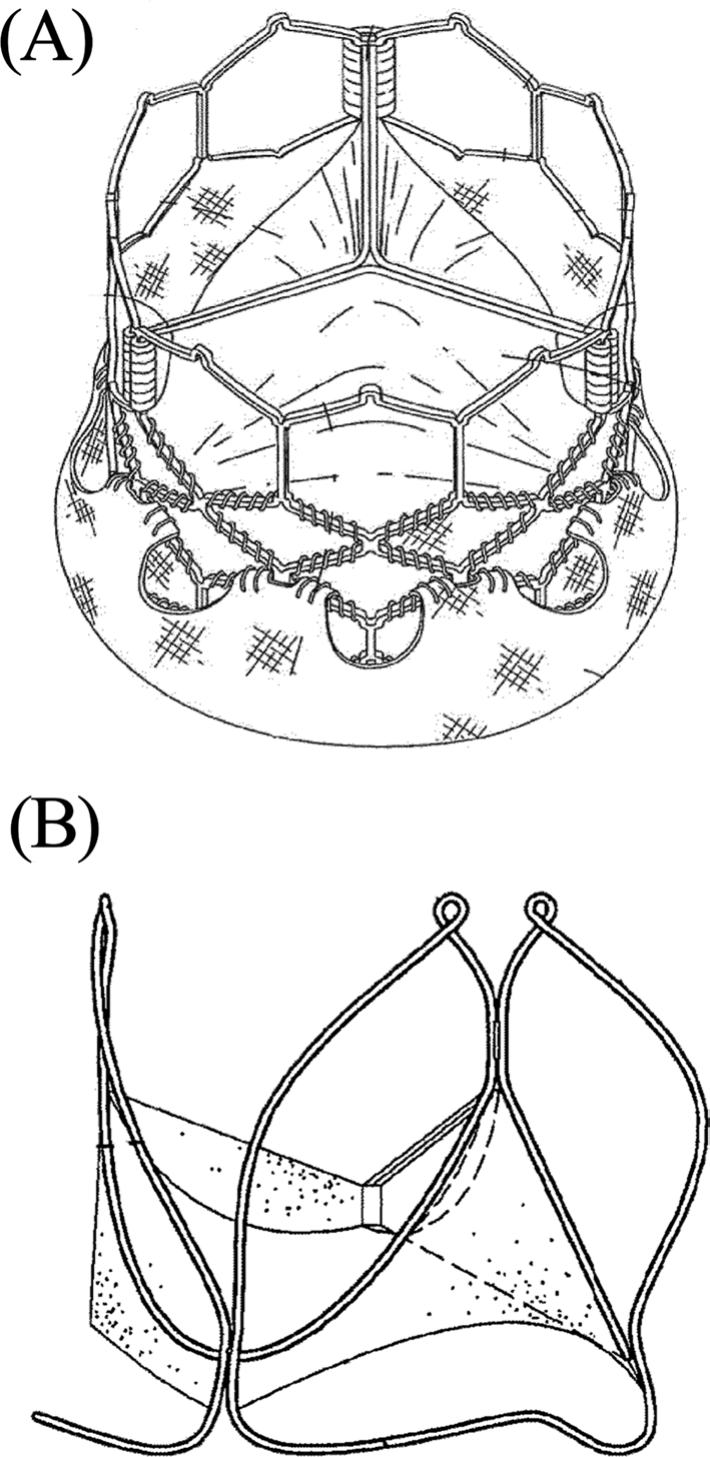
Schematics of different patented valve designs. **A** Prototype EP3485848A1—Courtesy of Edwards Lifesciences LLC, Irvine, CA. [40]. **B** Prototype US10357358B2—Reproduced with permission from Fondazione Ri.MED, Italy [41]

Patent EP3485848A1 (Fig. 3A), titled prosthetic heart valve, which is clinically used, consists of a stent, frame, valvular structure, inner skirt and an outer skirt. The three leaflets are built to collapse in a tricuspid arrangement. Choices of tissue that can be used to form the valve include the pericardial tissue (bovine) or biocompatible synthetic materials. The frame can be formed by any plastically-expandable materials (stainless steel) or self-expanding materials (nitinol). Once inserted into the patient, the frame (that has been crimped to a radially compressed state) can then be expanded by an inflatable balloon or similar expansion system. MP35N (a type of frame made with nickel–cobalt-chromium-molybdenum alloy) has better performance for radial and crush force resistance [40].

Patent US10357358B2 (Fig. 3B), titled heart valve prosthesis, published in 2019, comprises a support structure and a flow control structure. The support structure comprises a plurality of ribs. In the preferred embodiment, the ribs are made from metal wire, preferably of shape memory metal or superelastic materials, such as nitinol; however, they may be made of other materials, such as stainless steel or other deformable materials that are biocompatible or can be made biocompatible. The flow control structure of this embodiment or any of the other embodiments can be entirely synthetic, for example, formed from an artificial polymeric material, or can be biologically derived, for example, a xenograft of bovine pericardium or porcine pericardium, or a combination of synthetic and biologically derived [41].

Table 1 provides a summary of the prototype composition above, along with date, patent number and company responsible.

**Table 1:** Summary of the prototype composition, date, patent number and company responsible

Valve design patent number	Type	Company	Year	Composition	References
US20060271172A1	Prototype	Abandoned	2006	A valve body with a leaflet apparatus (using PTFE, bovine pericardium, or native porcine valve material) A supporting stent (metallic frame comprised of nitinol or stainless steel	[32]
US7846204B2	Prototype	Edwards Lifesciences Corp	2010	A valvular tissue made of a synthetic biocompatible material such as TEFLON or DACRON polyethylene, polyamide, or biological material such as pericardium, porcine leaflets	[33]
US20120165929A1	Prototype	St Jude Medical LLC	2012	The flow control structure of this embodiment can be entirely synthetic, e.g. an artificial polymeric material, or can be biologically derived, e.g. a xenograft of bovine pericardium or porcine pericardium, or a combination of synthetic and biologically derived	[34]
US8425593B2	prototype	UCL Business Ltd	2013	Buffer material or materials (e.g., polymer sheet or pericardial tissue sheet) are processed and cut to shape, and a bovine jugular or porcine aortic root (or individual leaflets) can be attached to the commissure posts of a valve stent	[35]
US8454685B2	Clinically used valve	Edwards Lifesciences Corp	2013	The frame can be made from nitinol to produce the 'self-expanding' valve function Alternatively, plastically expandable material that enables crimping of the valve to a smaller profile can also be used	[36]
US20140135911A1	Clinically used valve	Edwards Lifesciences Corp	2014	The valve assembly can be biological such as natural tissue, pericardial tissue or other types, and alternatively from biocompatible polymers or similar	[37]
US20140163667	Prototype	Speyside Medical LLC	2014	Ring-shaped support in the assembled valve which will be located at the base or annulus of the valve, as well as second ring-shaped support at the commissural region of the valve The shape of the hoops can be changed to a circular shape, a clover, or a semi-triangular shape to accommodate the natural aortic valve shape allowing a more uniform fit as well as less radial resistance in crush due to the irregular shape	[38]
US9532868B2	Prototype	St. Jude Medical Inc	2017	The leaflet structure is made of three flexible leaflet sheets composed of natural tissue, flexible polymer or similar. Each commissure post is partly cantilevered up towards the blood inflow, and the other post down towards the blood outflow	[39]
EP3485848A1	Clinically used valve	Edwards Lifesciences Corp	2019	A stent, frame, valvular structure, inner skirt and an outer skirt. The three leaflets are built to collapse in a tricuspid arrangement Tissues that can be used to form the valve are pericardial tissue (bovine) and biocompatible synthetic materials The frame can be formed by any plastically expandable materials (stainless steel) or self-expanding materials (nitinol)	[40]
US10357358B2	prototype	UCL Business Ltd	2019	A support structure and a flow control structure The flow control structure of this embodiment similar any of the other embodiments can be entirely synthetic, biological, or a combination of both	[41]

## Discussion

### Advantages of reviewed prototypes

Compared to older designs, the reviewed aortic valve prototypes contain advantages such as Bioprosthetic designs, minimal risk of embolisation, no anticoagulation requirement, absence of valve displacement, no risk of leakage or regurgitation, and minimal invasiveness [32–41]. Regurgitation or leakage was reduced through the use of an internal cover [33]. An additional advantage is the inability of the fibrous tissue to recoil, thus reducing displacement and preventing movement from the implanted position caused by the blood pressure changes with each contraction [32–34, 39]. Furthermore, an essential component of valves is the hyperboloidal shape which can maintain patency of the coronary ostia, which supplies the myocardial tissue with oxygenated blood. An open structure and smooth arc curvature of the 'ribs' in the valve frame decreases aortic root injury and disruption to blood flow [32–41].

In contrast, valve durability can be increased through a scalloped geometry, which reduces stress of the leaflets and enhances coronary sinuses’ perfusion [36]. Strain during crimping is also reduced through a U-shaped crown structure [36]. Additionally, using MP35N, a nickel–cobalt-chromium-molybdenum alloy, for the frame further increases radial and crush force resistance. Reduced relative movement between the valve assembly and the support beams also plays a vital role in increasing durability during stress environments. Strong stress points are prevented by avoiding suture requirements on the working leaflet. High radial strength increases the ability of preventing restenosis in severely calcified aortic valves [36, 38]. Whereas achieving a smaller parameter or diameter design of the folded or crimped position improves the delivering ability while allowing a greater selection of patients to be treated [39, 40]. Ex-vivo collapsibility before implantation is a significant advantage as this does not require a specialist compression tool, therefore increasing the speed of the implantation process [34, 39].

Table 2 provides a comparison of the similarities between the prototype and clinically used valve designs reviewed and the ideal valve type.

**Table 2:** Comparison of similarities between the prototypes and the ideal valve

Valve design patent number	Similarities to ideal valve	References
US20060271172A1	Three leaflet apparatus A superior and inferior O-ring assembly which prevents leakage around the valve, thereby increasing the efficiency of the valve	[32]
US7846204B2	Three leaflet apparatus Internal cover that prevents regurgitation, thereby increasing the efficiency of the valve Movement of blood is not hindered, due to the hyperboloidal shape design which preserves valve efficiency	[33]
US20120165929A1	Three leaflet composition Open structure and smooth arc curvature of the ribs prevents disruption to blood flow and injury to adjacent tissue, therefore preserving the efficiency of the valve Less susceptible to clotting, reducing the risk valve efficiency being affected	[34]
US8425593B2	Three leaflet composition Annularly collapsible and re-expandable support structure, which reduces stress on the leaflets, therefore preserving the leaflet structure Zero folds in the leaflets (equal length of the leaflet free edge in systole and diastole), thereby cancelling any stress due to folding	[35]
US8454685B2	Three leaflet composition By reducing stress through the scalloped geometry of the leaflets, durability is preserved, and valve efficiency is maintained Reduced strain during crimping due to the U-shaped crown additionally preserves durability and ensures that valve efficiency is maintained	[36]
US20140135911A1	Three leaflet cusps Does not have relative movement between the valve assembly and the support beams which ensures durability and valve efficiency Absence of requirement of sutures, therefore preventing concentrated stress points on the leaflets, and therefore improved valve durability	[37]
US20140163667	Ability to withstand restenosis and high radial strength which increases efficiency in severely calcified aortic valves Accommodates the natural aortic valve shape for optimal performance	[38]
US9532868B2	Three flexible leaflet structure Each commissure is centilevered up towards the blood inflow and the other post down towards the blood outflow, therefore ensuring efficiency of blood flow through the valve Improved stress absorption from the lateral edges of the leaflets Prevention of paravalvular leakage by the extra sealing effect produced by the toroidal section of the cuff, thereby increasing efficiency of valve function	[39]
EP3485848A1	Three leaflet composition Smaller parameter, enabling a greater patient selection and possibly minimal valve leaflet flexion and height Reduced stress and on the leaflets due to the curved scalloped geometry of the leaflets	[40]
US10357358B2	Decreased effects on the adjacent tissue and minimal disruption to blood flow, due to the open structure and the smooth arc curvature of the ribs Less clotting risk therefore improved durability and efficiency	[41]

### Limitations of reviewed prototypes

Despite the advantages, the reviewed valve prototypes contain limitations when compared to the ideal valve type. Limitations include compatible replacement after a couple of years due to degeneration, thrombotic risk in synthetic designs which require long term anticoagulation use and destruction of the flow structure due to early compression of the valve [32–41]. Delaying the collapsing of the valve until before the insertion prevents the destruction to the flow structure [39]. However, postponing the collapse may result in logistical problems. Furthermore, breakage and collapse of the valve structure can occur following anchorage during the drawing process, suggesting that further improvement is required in the delivery system [39]. Several designs were more limited by peri-valvular leakage, mitral valve impingement from deployment that was too deep into the left ventricle or positioning too high into the aorta and low long-term durability [32–35, 37]. Some designs with scissor-like motion of the frame struts increased concern, as pinching of leaflets could occur between inner surface of the metal frame and struts during the motion [36]. Additionally, severe crimping of the valve to achieve a small crimping size may result in cuts and rupture of the tissue leaflet [36].

Improvement in the ability to measure the 'pure' stressed state is still required, as the current 'creep measurement' only calculates the constant stress which does not portray the deflection that occurs in the specimen during the normal valvular function [25]. High forces that are exerted on the four-layered commissures such as the mounting process onto the delivery shaft may result in tearing of the four-layered commissures [25]. The outward protrusion can equally occur if the valves are mounted too close to the distal end of the frame [25]. Multiple features of the ideal valve that were not clearly stated or addressed by the prototypes patents include minimum coaptation height which ensures the safety of closure and efficiency of the valve, the absence of folds in the leaflets together with equal length of the leaflet free edge in systole and diastole [25]. The absence of folds cancels out additional forms of stress experienced by the valve [25]. Furthermore, features that were not addressed in the patents include minimum valve height, minimum leaflet flexion, lowered dead space and flexion stress, which preserve the energy of the leaflet by decreasing its motion [25, 32–40].

### Thrombogenicity aspects and its association with the prototypes

A study comparing the thrombogenic potential of 55 laboratory constructed prototypes against tissue valve controls manufactured by St Jude Medical, and Edwards, found that controls were outperformed by a factor of three in both aortic and mitral regions [42]. Reducing the closure related velocity aided the achievement of decreased thrombotic outcomes. Indicating that the bi-valvular prototypes manufactured by St Jude Medical and Edwards are predisposed to greater thrombotic risk due to higher valve closure related velocities. However, our paper only addressed tri-leaflet prototypes. Another study showed reduced thrombogenicity in tri-leaflet polymeric prosthetic valves compared to other valves [43]. Patents US20120165929A1, US8425593B2 and US9532868B2 therefore appear to be superior in reducing thrombogenic occurrence due to the option of having a polymeric valve material (Table 1). Furthermore a case report has recorded 14-cases of valvular thrombosis with the Edwards-SAPIEN design in other studies [44]. Although, the primary cause for the thrombosis incidence was insufficient prescription of post-op anticoagulation rather than the valve design itself. In addition to these findings, Kounis syndrome, a recent term used to describe allergic reactions resulting in acute myocardial events, can be suggested as a likely explanation for valve predisposition to thrombis formation [45]. Stent material thought to result in myocardial sensitisation and therefore thrombus formation include nickel, titanium, molybdenum and iron [46]. Both the patents EP3485848A1 and US20060271172A1 have the option of using nitinol for the stent frame (Table 1). Despite the later prototype being an abandoned design, patent EP3485848A1 is still in use clinically. Consequently, patent EP3485848A1 has the greatest predisposition to Kounis Syndrome.

## Conclusion

As of today, and based on the discussion, the prototype that is closest to the ideal with the most similarities is the prototype, collapsible-expandable prosthetic heart valves with structures for clamping native tissue, from the year 2017 (Table 2). In this paper, our subjective understanding is being portrayed of the ideal valve. We understand that there are other features to be considered, which can be found in the paper written by Figulla et al. [47].

Based on what was discussed and analysed, an ideal Aortic valve must have a minimum coaptation height which ensures safety and proficiency during closure of the valve. Zero folds in the leaflets cancels out the stresses due to leaflet folding, by making the length of the leaflet free edge in systole equal to the length of the leaflet free edge in diastole. The ideal valve also requires a minimum valve height to lower the dead space, as well as minimum leaflet flexion, to lower the flexion stresses and preserve the energy by decreasing the motion of the leaflet to a minimum. Additionally, the ideal aortic valve must be composed of three leaflets [25]. And finally, the ideal valve must have the lowest risk of thrombosis, to avoid long term complications and medication use. Reduced risk of post-operative thrombosis can be achieved by reducing the valve closure velocity in mechanical bi-valvular designs [42]. Tri-leaflets can also achieve reduced thrombosis risks by using polymeric designs [43]. Further complicating the predisposition to thrombosis of newly designed valves, is the potential for an allergic reaction to the valve material itself, i.e. Kounis syndrome [45]. Currently, literature primarily describes patient co-morbidity and anti-coagulant use as having the predominant role in affecting thrombosis risk. Nevertheless, literature still lacks research that targets valve designs and their likelihood towards causing thrombotic events. These factors contributing to the construction of the basis of the aortic model are significant. However, more studies and analysis about the material used and the parameters discussed are needed to apply the model for different aortic valve diseases and anatomies [48]. The need for further investigations is due to the different features required based on the case, need and preference of the patient.

## Data Availability

The datasets used and/or analysed during the current study are available from the corresponding author on reasonable request.

## Abbreviations

TAVR: Transcatheter aortic valve replacement
PET: Polyethylene terephthalate
PTFE: Polytetrafluoroethylene
